# In-silico design of computational nucleic acids for molecular information processing

**DOI:** 10.1186/1758-2946-5-22

**Published:** 2013-05-07

**Authors:** Effirul Ikhwan Ramlan, Klaus-Peter Zauner

**Affiliations:** 1 Department of Artificial Intelligence, Faculty of Computer, Science and Information Technology, University of Malaya, 50603 Kuala Lumpur, Malaysia; 2 ECS, Faculty of Physical and Applied Sciences, University of Southampton, Southampton, SO17 1BJ, UK

**Keywords:** Functional nucleic acids, Ribozymes, Nucleic acids computer, Molecular logic gates

## Abstract

Within recent years nucleic acids have become a focus of interest for prototype implementations of molecular computing concepts. During the same period the importance of ribonucleic acids as components of the regulatory networks within living cells has increasingly been revealed. Molecular computers are attractive due to their ability to function within a biological system; an application area extraneous to the present information technology paradigm. The existence of natural information processing architectures (predominately exemplified by protein) demonstrates that computing based on physical substrates that are radically different from silicon is feasible. Two key principles underlie molecular level information processing in organisms: conformational dynamics of macromolecules and self-assembly of macromolecules. Nucleic acids support both principles, and moreover computational design of these molecules is practicable. This study demonstrates the simplicity with which one can construct a set of nucleic acid computing units using a new computational protocol. With the new protocol, diverse classes of nucleic acids imitating the complete set of boolean logical operators were constructed. These nucleic acid classes display favourable thermodynamic properties and are significantly similar to the approximation of successful candidates implemented in the laboratory. This new protocol would enable the construction of a network of interconnecting nucleic acids (as a circuit) for molecular information processing.

## Background

Early suggestions for implementing a molecular computer with nucleic acids followed the encoding principle of genetic information [1]. This would require the formation and cleavage of numerous covalent bonds for their operation and thus require specific sets of enzymes. Major progress in the application of nucleotides for information processing came about two decades later with Adleman’s insight that random oligonucleotides could be the basic tokens for information processing [2]. His method employed enzymes only to stabilise (through covalent bonds) the products of a self-assembly process (hybridisation of partially complementary oligonucleotides) but not in the information processing itself, and accordingly did not require enzymes with sequence specificity. This implementation by Adleman spawned the idea of building nucleic acid (DNA) computers as an alternate computing means that possess greater computational power than the conventional machines. However this view has changed, as currently nucleic acids computers are being designed and engineered to function inside a living cell [3-7].

The discovery of short RNAs’ (which are 21–25 nt in length) role in regulating gene expression [8] has sparked a strong interest in RNA molecules. For instance, in RNA interference, a short interfering RNA molecule (siRNA) forms complementary base pairs with a target region in mRNA, allowing a protein complex called RISC (RNA-induced silencing complex) to attach and cleave the target region [9,10]. Another short RNA called microRNA (miRNA), generated from an enzyme named Dicer that cleaves non-coding RNAs (i.e., RNA that do not code protein), binds imperfectly with the target region in mRNA (forming a bulge), thus preventing the translation machinery from accessing this target region [11]. In addition to these short RNAs, another complex folded RNA domain called riboswitch also plays a role in gene regulation. Riboswitches sense the presence of specific metabolites and harness their conformational switching to activate the gene-control mechanisms in preventing the production of protein [12,13]. The ability of these RNA molecules provides an interesting application scenario for molecular information technology.

### Nucleic acids as substrate for information processing

**Figure 1:**
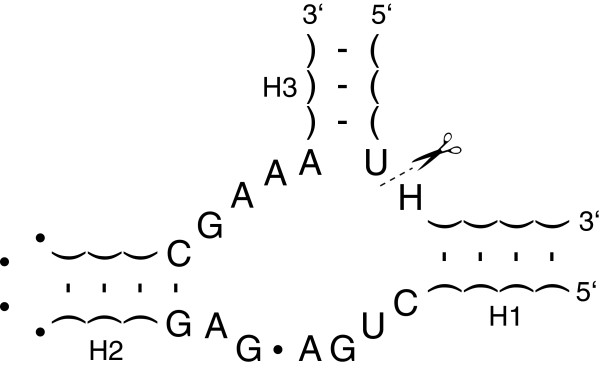
**Minimal functional structure of hammerhead ribozyme.** Three helical stems (H1, H2, H3) emanate from a junction on the ribozyme core [17,18]. In nature, either helix H1 or H3 is terminated by a hairpin loop, which results in intra-molecular catalysis. Hammerhead ribozymes that catalyse the *in-trans* reaction, as depicted in the figure, can be made synthetically [19]. The core region has a specific sequence for all known active structures and is therefore termed ‘conserved’. Conserved bases are specified explicitly, with H representing any one of {A, C, U}. A dot (∙) stands for any base that will not cause hybridisation in this position; correspondingly two parentheses connected by a dash indicate an arbitrary pair of complementary bases. Hammerhead ribozyme cleaves the substrate strand that binds to form H1 and H3 as symbolically represented by the scissor and dashed lines.

**Figure 2:**
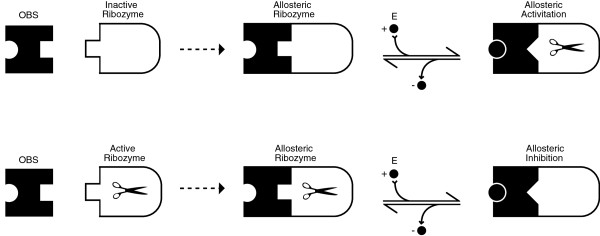
**Allosterically controlled ribozymes.** Allosterically activated ribozyme (top) and allosterically inhibited ribozyme (bottom) [20,21]. The allosteric ribozyme is composed of two components (left of the dashed arrow), a oligonucleotide binding site (OBS) and a ribozyme part. The two components are covalently bound and form a single nucleic acid molecule (centre). Upon binding an effector oligonucleotide (E) the conformation of the binding site changes and affects the conformation of the ribozyme component. The latter conformational change will activate (top) or inhibit (bottom) the catalytic activity of the ribozyme part. The same scheme can also be realised with deoxyribozymes. The scissors symbolically represent the cleavage reaction of the ribozyme.

Computational nucleic acids are constructed as modular tuneable units, where different components of one system can be substituted with alternative parts as demonstrated in proof-of-concept models of simple computational units [14,15] and as synthetic devices integrated within living cells [4-6]. These proof-of-concepts are founded by a particular type of RNA molecules, so called *ribozymes*, that can act as catalysts. Moreover, the catalytic activity may be enabled or suppressed upon binding of a nucleotide strand [16]. It is even possible to design and fabricate ribozymes endowed with multiple interacting effector binding sites. On the one hand, the combinatorial variety of nucleic acid strands allows for the numerous different effector molecules and accordingly facilitates the independent parallel operation of several allosterically controlled ribozymes. On the other, the fact that such ribozymes can have the same type of molecules, i.e., RNA oligonucleotides, as effectors and as products of the reactions they catalyse opens up a path to cascading several processing stages for molecular signals. Within the context of molecular information processing, a ribozyme called *hammerhead* appears to be the most suitable as the enyzmatic core for the construction of allosterically controlled ribozymes (The secondary structure of a hammerhead ribozyme is depicted in Figure 1). The concept of allosterically controlled ribozymes is illustrated in Figure 2.

Input signals are encoded as small molecules of DNA strands which effect a computing machinery that combines both functional nucleic acids (ribozymes for RNA and deoxyribozyme for DNA) and receptor units for input signal detection. The key to this approach lies in the possibility of controlling the activity of a ribozyme or deoxyribozyme with oligonucleotides as input. Such allosterically controlled nucleic acid enzymes have been investigated as sequence specific biosensors, where they have the advantage over molecular beacons because they can catalytically amplify the recognition event [22]. Within certain constraints, the base sequence for the binding site of the control oligonucleotide (labelled OBS in the Figure 2) can be chosen independently of the sequence on which the nucleic acid enzyme will act. It is therefore possible to have an oligonucleotide sequence start (or stop) the production of another, largely independent, oligonucleotide sequence. Moreover, it is possible to engineer nucleic acid enzymes to be controlled by more than one oligonucleotide. It is even possible to design and fabricate ribozymes endowed with multiple interacting effector binding sites.

The tuning of the computational units (i.e., ribozyme and the receptor sites) is achieved by altering the sequence of bases in certain regions of the nucleic acids guided by energetic information that can be calculated from the sequence to structure mapping of the molecules. In a similar manner, one can allocate sequence constraints that can enhance sequence specificity for any particular part of the computational units. In designing sets of nucleic acids for information processing, one typically has a desired molecular conformation and additional local constraints specific to certain regions of the molecules. For example, a binding site for an effector molecule (i.e., a nucleic acid that will affect the activity of a functional nucleic acid) may be required to be complementary to a sequence released in a preceding step.

Following this approach, one is likely to construct simpler information processing units that can be integrated into a network, where output from one unit can be used as input for another corresponding unit. The *in-vitro* demonstrations of such networks have been shown by Penchovsky and Breaker [15] and Stojanovic et al. [23]. The nucleic acid constructs are employed to solve simple arithmetic operations [24,25] and capable of handling tasks that require the integration of several different types of molecular gates with a common set of input and substrate molecules [14,26]. Thus far, the construction of a network comprising more than 100 nucleic acid molecular gates has been reported [27], suggesting that, the development of highly regulated molecular networks able to support complex decision-making criteria is feasible.

In a conventional machine, the components (i.e., hardware) are wired according to a predefined transfer function (e.g., a logic circuit is mapped to “YES” given a state of x element as “YES”) [2]. In this paradigm, the set of actions are already predetermined resulting into a finite set of input and output mapping (i.e., a large and complicated hash-map table). The construction of conventional logic gates as a molecular computer however, does not imply that logic gates are a viable strategy for implementing computational nucleic acids. Nevertheless, if a nucleic acid circuit is to be constructed for the purpose of regulatory control, then, the design of binary logic operators used here as a test case has a direct application.

Although there exists various nucleic acid computers, the basis (in which each unit functions or operates) remains the same. By utilizing self-assembly to bind to the respective receptor regions, a chemical reaction is instigated, causing the molecule to undergo conformational changes. These reactions will either release an output signal or trigger consequent self-assembly reactions to de-form the molecules into different states (i.e., conformational change).

## Methods and materials

### Constructing the RNA molecular “PASS” logic gates: A retrospective

In contrast to proteins, there exist well established computational tools that can aid in the secondary structure prediction and sequence design of RNA molecules. By combining *RNAfold* for secondary structure prediction, *RNAinverse* for sequence design, *Kinfold* for simulating the kinetic pathway of a secondary structure folding (for an RNA sequence), and *RNAcofold* for measuring the efficiency of intermolecular binding (from the Vienna RNA package of [28]), Penchovsky and Breaker [15] derived a computational protocol to construct RNA logic gates using the allosterically controlled ribozymes architecture. The protocol of Penchovsky and Breaker [15] however, imposes strict structural constraints during the design process in an effort to produce only plausible candidates to undergo the in-vitro selection process. However, these structural constraints limit the ability of the protocol to produce different structural designs, which are required in order to construct computational nucleic acid circuitries comprising of hundreds of interacting molecules.

The simplest logic gates have only one bit input. The NOT gate that inverts the input and the PASS gate (sometimes also called “identity” or YES gate) that forwards the input signal. Although, from a purely logical viewpoint PASS gates serve no purpose, in practise they can reform a degraded signal or adjust signal delay [29]. The molecular pass gates considered here are more powerful than one-bit logic gates and an essentially arbitrary input sequence of limited length can be recoded into a different output sequence.

In Ramlan and Zauner [30], using a variant computational protocol (in this paper referred as P-ER1) developed from the original suggested by Penchovsky and Breaker [15], we have demonstrated the possibility of producing more diversified structural configurations for the molecular PASS gates, which are quite unique, compared to the homogeneous configurations implemented in the original protocol. Our protocol for designing an allosterically controlled ribozyme for one-input logic gate comprises of three generating steps, (the same steps outlined in Table 1 with only a single effector binding region instead of two as depicted in the table) to arrive at a sequence design. Sequence generation is followed by a series of validation steps.

**Table 1:** Proposed computational protocol for designing two-input molecular gates

	Randomisation of lengths for the extension region that is comprised of a helix that attaches to the ribozyme core, two effectors binding regions or OBS (NN ⋯NN), and three linker sequences connecting the binding sites with the helix (inside dotted box) are generated. Parameters are restricted to those given in Table 6.
	Sequence positions except the binding sites are assigned by searching for a base sequence that will fold into the target structure designed in the previous step using *RNAinverse*[28] or *RNAdesigner from RNAsoft*[31], with a non-binding pseudo-base (N) being assigned to all positions in the binding region.
	Replacement of the pseudo-bases in the binding regions with real bases. All possible combinations of effector binding are considered using (NN ⋯NN) pseudo-bases to represent an unoccupied binding region. This can be verified with *MFOLD*[32] or *RNAfold from Vienna*[28].

Proposed computational protocol (P-ER2) for designing two-input molecular gates. This revised procedure differs from the P-ER1 protocol to design molecular PASS gates presented in [30] by adding another effector binding site and a linker in the extension region of helix II.

Although our protocol (P-ER1) successfully increases the degree of freedom in designing the molecular PASS gate, however we acknowledge that the variant computational protocol is far from being efficient. There is an increase in computational time and a significant decline in the number of solutions plausible for laboratory implementation(based on the filter cascade suggested by Penchovsky and Breaker in [15]). Evidently, the increase in the structural space has a decremental effect towards the generation of quality candidates (to be evaluated in the laboratory). A compromise between promoting structural diversity and the generation of plausible candidates must be addressed in order to ensure a successful implementation of computational nucleic acids.

### Constructing the complete set of RNA molecular logic gates: Conventional RNA sequence designer

**Figure 3:**
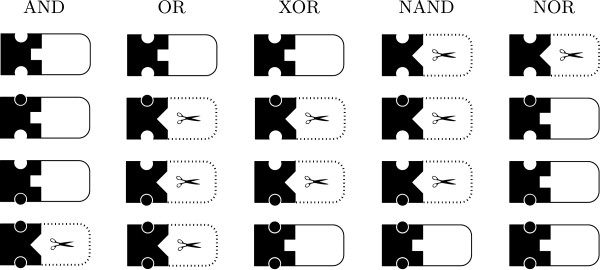
**The abstract representation of RNA molecular logic gates.** The abstract representation of RNA molecular logic gates with (AND, OR, XOR, NAND and NOR) operations. The shift in the conformational dynamics is indicated by the change of the rectangle into a triangular shape and the change of the solid line curves into dotted lines. Catalytic activity is marked by the scissors symbol.

In this section, we discuss the design of nucleic acid molecular gates that follow the logic of all possible two inputs conventional logic gates. The complete truth table of all the possible two input logic gates is listed in Table 2. In contrast, Figure 3 illustrates the abstract representation of the nucleic acid logic operators.

**Table 2:** Two-input binary logic gates

**Line**				** Input state**		
A	1	0	1	0		
B	1	1	0	0		
**Operation**				** Output state**	**Name**	**Symbolic**
A ∘_1_B	0	0	0	0		0
A ∘_2_B	0	0	0	1	NOR	A ◇ B
A ∘_3_B	0	0	1	0		
A ∘_4_B	0	0	1	1	NOT B	¬ B
A ∘_5_B	0	1	0	0		
A ∘_6_B	0	1	0	1	NOT A	¬ A
A ∘_7_B	0	1	1	0	XOR	
A ∘_8_B	0	1	1	1	NAND	A ∣ B
A ∘_9_B	1	0	0	0	AND	A ∧ B
A ∘_10_B	1	0	0	1	Equivalence	A ⇔ B
A ∘_11_B	1	0	1	0		A
A ∘_12_B	1	0	1	1		
A ∘_13_B	1	1	0	0		B
A ∘_14_B	1	1	0	1	Implication	A ⇒ B
A ∘_15_B	1	1	1	0	OR	A ∨ B
A ∘_16_B	1	1	1	1		1

Two-input binary logic gates.

Instead of the binary operation of conventional logic gates, biomolecules offer richer operations. For instance, a molecular gate can be made from an allosterically controlled hammerhead ribozyme, where one can attach different substrate strands to be released as output sequence, and design the receptor sites with different effector molecules as inputs [15]. The substrate is cleaved when the ribozyme is activated, while the effector binds to the receptor site to steer conformation change that activates the ribozyme. However, it is possible to design a substrate strand that can function as an effector molecule for another allosterically controlled ribozyme, for instance in the design of a cascade of nucleic acid computers. The substrate and effector molecules may have different sequences, therefore an RNA AND gate, although it follows the logic AND operation (i.e., only releases its output when both effector molecules are present) does not directly correspond to a conventional AND gate.

The two-bit input and one-bit output signals of the conventional logic gates are represented by essentially arbitrary nucleotide sequences. In principle, there are 4^*n*^ base combinations for the input and output molecules, where *n* denotes the length of the signalling molecule. Two or more molecular gates that are common in their activation mechanism could be completely different in terms of their structural design and mechanism [15]. If a cascade of logic gates is to be developed, the dynamics of the molecular logic gates not only allows for the use of output strands as effectors but also as substrates in subsequent processing stages.

From the computational protocol P-ER1 for the design of PASS gates, we derived a variant protocol named P-ER2 that will allow the search for the logic operators as listed in Table 2 by adding another effector binding site and linker to the extension region of helix II. As in the P-ER1 protocol, we initially start with an active hammerhead ribozyme configuration, then, search for a sequence combination to be placed in the effector binding region that distorts the active hammerhead motifs. But in the case where two effectors are required, we extended the protocol to check for all four possible meta-stable conformations of the molecule (i.e., [no E], [E_1_], [E_2_] and [E_1_ and E_2_], where E denotes the effector molecules). Table 1 depicts the protocol of P-ER2 for the design of two-input molecular logic gates.

In order to increase the probability of generating sequences that will disrupt the formation of the hammerhead motifs, the complementary bases of the conserved region are embedded at arbitrary locations within the linker extensions, or at the effector binding sites, or overlapping both. Details on various strategies to design allosterically controlled functional nucleic acids (specifically hammerhead ribozyme) are discussed in [30].

There are two conditions that need to be investigated in order to generate sequences for the complete set of logic operators. Based on the binary logic table, represented in Table 2, we start searching from the structures that are active with the presence of both effector molecules (i.e., the normal direction of the previously described protocol–cf. Table 1) and secondly, starting from the reverse direction, where the presence of both effectors does not affect the inactive state of the ribozyme (i.e., the conserved regions of hammerhead ribozyme are bound randomly at the start of the search). By fulfilling the first condition, we can generate sequences for the bottom half of the binary logic function as depicted in Table 2, where in the presence of both effector molecules (input-A and input-B in the table) the catalytic function is activated, and by satisfying the second condition, we can produce solutions for the top half of Table 2, where in the presence of both effectors the catalytic function is always deactivated. For clarity, the logic operators for the first condition are referred to as LG-B, and the logic operators for the second condition are referred to as LG-T.

We first investigated the distribution of two-input gates that are generated by the automatic design protocol (P-ER2). For this purpose 50,000 candidate sequences were generated using the protocol. The results are as shown in Table 3. Any response pattern of the generated structures to two effector molecules will correspond to a row in Table 2. However, the top row (A ∘_1_B) and the bottom row (A ∘_16_B) correspond to the case of a constant OFF output and a constant ON output. These two cases that ignore the effector molecules entirely will not be considered further. Only ≈43% of the total candidates can be classified as imitating the conventional binary logic operation where as the remaining ≈57% fall directly under the constant 0’s and 1’s logic operators, with the latter forming the majority ≈95% of the constant gates.

**Table 3:** Distribution of candidate sequences generated by the P-ER2 computational protocol

	**Input state**							**Input state**												
A	1	0	1	0			A	1	0	1	0		
B	1	1	0	0			B	1	1	0	0		
		**Output state**			**Total**	**Success**		**Output state**				**Total**	**Success**
		**(LG-B)**			**candidates**	**rate**		**(LG-T)**				**candidates**	**rate**
	1	0	0	0	1517	5.4%		0	0	0	1	1006	42.8%
	1	0	0	1	326	26.0%		0	0	1	0	3276	26.5%
	1	0	1	0	127	11.0%		0	0	1	1	786	1.7%
	1	1	0	1	167	0.0%		0	1	0	0	3348	29.3%
	1	1	0	0	10004	8.9%		0	1	0	1	1194	3.9%
	1	1	0	1	7470	2.7%		0	1	1	0	200	18.5%
	1	1	1	0	1850	16.6%		0	1	1	1	200	0.0%
		Constants			28539	-		Constants				39990	-

Distribution of candidate sequence generated by the revised computational protocol (P-ER2) in Table 1, classified according to the type of binary logic operator LG-B (depicted in the bottom half of Table 2) and LG-T (depicted in the top-half of Table 2). For LG-B, it is mandatory for the operator to be active, whenever the two inputs are present. Accordingly, for LG-T, The initial start of the design requires the molecule to remain inactive despite the presence of both inputs (i.e., reverse scenario of LG-B). The candidates generated at each run will then undergo a filter cascade (cf. Table 4). The total candidates column represents the total number of candidates generated by the protocol. The percentage of sequences that passed the cascade is listed in the success rate column.

**Table 4:** Filter cascade for candidate sequences

**Stage**	**Filter**	**Condition to satisfy**
1	Identical nucleotides	No more than three identical consecutive nucleotides in the oligonucleotide binding site(s)
2	Active state conformation	The formation of an active hammerhead conformation based on the truth table condition (cf. Table 2)
3	Base-pairing percentage	In the absence of effector(s) 30%–70% of the oligonucleotide binding region is hybridised
4	Energy gap	Energy gap between the inactive and active state is within -6 kcal/mol to -10 kcal/mol
5	Temperature tolerance	Structure is preserved over a temperature range of 20°– 40°C
6	Ensemble diversity	For neither active nor inactive state the ensemble diversity (cf. [33]) exceeds 9 units
7	Folding efficiency	The RNA molecule must fold, in the absence of the effector, to the inactive conformation within 480 units in *Kinfold*[34].

Constraints imposed on candidate sequences following [15].

This indicated that the conformation of the ribozyme core remains active despite the absence of the effector molecules, and subsequently, remain unaffected with the presence of either one or even both of the effector molecules. This is equivalent to the failure of allocating a base pairing region for the conserved bases during the design of the effector binding region (OBS), which is intended to disrupt the catalytic activity of the ribozyme core.

As shown in Table 3, the P-ER2 protocol, with the first search strategy (LG-B), managed to generate candidate sequences for the logic operators in the bottom half of Table 2 (LG-B). However, as indicated in the “Success Rate” column, after the filtering process (i.e., this filtering process was adapted from Penchovsky and Breaker’s [15] protocol with the same objectives, which is to eliminate candidates unsuited for *in-vitro* implementation. This filtering process is summarised in Table 4), we observed a significant decrease in the number of candidates for each classified operator. Upon closer inspection, the passing percentage reduced significantly during the execution of filter steps 3 and 4. As we are removing some of the constraints in the structure specification, in order to increase the degree of freedom and the space of plausible structure configuration, the decrease in candidates occurring in both filters are inevitable, for a procedure that relies on the single state sequence design algorithm in generating its candi- date sequences. The ideal solution would be to replace the single state design algorithms with a multi-stable design algorithm, which minimises the energy gap between the meta-stable states.

Next we focused on the search for the LG-T binary logic operators, where the operator would remain inactive, in the presence of both input molecules (cf. top half of Table 2). For the second design strategy (LG-T), the protocol (P-ER2) assigns the complementary base pairs of the ribozyme core (conserved CUGAUGAG-region) in any random positions within the extension region of helix II (cf. figure panel in Table 1).

From Table 3, with the exception of three gates (where the success rates are lower than ≈4%), the success rates of the remaining logic gates are slightly better than the success rate for LG-B. Despite the slight increase in the success rates, more than 75% of the candidates are discarded during the filtering stages. However, the candidate sequences of LG-T have a higher passing rate in filter step 4 compared to the generated candidate sequences of LG-B, although we observed that the passing rate in filter step 3 is still mediocre. The results indicate, that the computational protocol (P-ER2), which aims to increase the degree of freedom in generating a diversified structure configuration for the design of molecular logic gates seems to be rather inefficient. The meta-stable conformations of the molecules are not considered during sequence assignment by the single state sequence design algorithm (panel 2 in Table 1). Instead of the single state sequence design algorithms, for the sequence assignment of computational units with multi-stable conformations, a multi-stable sequence design algorithm is required.

### Constructing the complete set of RNA molecular logic gates: Multi-stable sequence design algorithm

From the engineered DNA and RNA logic gates [14,15] to the development of synthetic RNA devices [5-7,35], the type of molecules that are of interest for information processing tasks have a number of meta-stable conformations representing their change of folding with regard to the changes in their environments. For instance, an allosterically controlled hammerhead ribozyme imitating the AND logic operator [15] has four different meta-stable conformations. These conformations are representative of four conditions, i.e., when no effectors are present, when one effector is present but not the other, and when both effectors are present. For naturally occurring riboswitches, there could be a number of possible meta-stable conformations which includes the binding of specific metabolites to their receptor sites and the different stages of conformational shift to triggers their gene control mechanisms, as discussed by Nudler [36] and Suess and Weigand[37]. Therefore, in order to design nucleic acid computing units, a protocol that includes a multi-stable sequence design algorithm is required. The development of such protocol is discussed in this section, and using RNA logic gate as test case, we evaluate the performance of the new protocol.

From our detailed analysis on the results generated in Table 3, we identified that the pool of candidates were significantly reduced during filter step 3 (base-pairing percentage) and 4 (energy gap). It appears that the use of a single state sequence designer is insufficient, largely because the multi-stable characteristics (i.e., conformation, free energy and energy gap) are not part of the optimisation objective of the single-state design algorithms. There is a need to substitute the single state sequence designer with a multi-stable sequence design algorithm to take into account the multi-stable characteristics of the molecules. The reduction in filter step 4 is related to the method of constructing the initial structural configurations of the molecule (step 1 in Table 1). We showed that a less restrictive space is required to produce a diversified set of molecular gates, but accordingly the design space should be constructed by considering a few essential design elements of the molecular gates, e.g., the placement of the receptor sites and the base pairing complementary of the conserved region.

Using the P-ER2 protocol (Table 1) as a basis, we derived a new protocol named P-ERM, which includes *multiSrch*[38] as the multi-stable sequence design algorithm that replaces the single state design algorithm used in P-ER2. Because of the ability of *multiSrch* to generate sequences for all interacting molecules, we are able to combine the last two steps of P-ER2 (middle and bottom panels in Table 1) into a single step. The result is a simplified two-step protocol (Table 5): firstly we create the plausible structure configuration (known as “partial conformation”) representing the molecules based on their structural and sequence constraints, and secondly the generation of sequences using *multiSrch* with the meta-stable partial conformations(defined in the first step) as input.

**Table 5:** Computational protocol (P-ERM)

**Figure 4:**
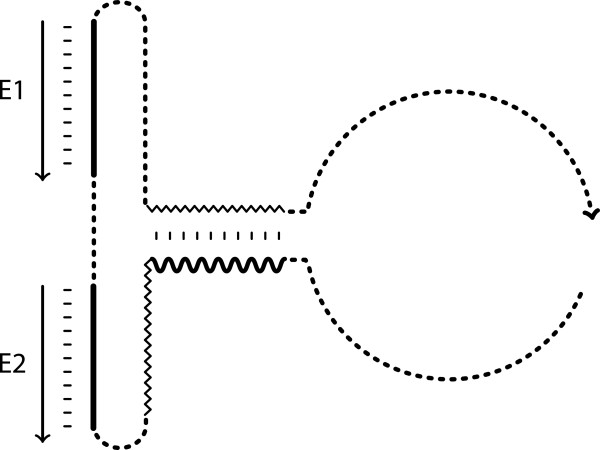
**A sample structure configuration depicting a partial conformation of the binary logic XOR operation.** A sample structure configuration depicting a partial conformation of the binary logic XOR operation, where the presence of two effectors yield no activation of the molecule (cf. XOR in Table 2). The crinkled lines in the figure represent the helix II base pairing of the active hammerhead ribozyme, while the bold wavy line represent conserved region (CUGAUGAG). The small dashed-lines denote un-fixed conformation, where the base position belonging to this region can be either paired or unpaired as long as other mandatory regions (i.e., the base pairing of CUGAUGAG-region and the complementary half of the helix II, effector 1 (E1) with its binding site and effector 2 (E2) with its binding site) are present.

	Generate all “partial” meta-stable conformations (refer Figure 4 for details) for the molecules. The length of the receptor sites or OBS (bold lines), the helix II (short crinkled lines), and the linkers (cf. Table 1) are randomised within the constraints detailed in Table 6. For instance, to design an XOR gate, four meta-stable partial conformations are provided. Two inactive states where the hammerhead ribozyme motif is distorted (top left and bottom right) and two active states where the hammerhead ribozyme motif is formed when only one of the effectors is present (top right and bottom left).
	Using *multiSrch*[38], generate sequences that conform to the meta-stable conformations from the previous step. Bases are assigned for positions in the bold regions.

Computational protocol (P-ERM) for designing two-input molecular gates based on a multi-stable sequence design algorithm (*multiSrch*) developed in [38].

In P-ERM protocol, the generation of structure in the first step is not entirely random as in the previous protocols (P-ER1 and P-ER2). Only the variable lengths of the regions were arbitrarily selected (cf. Table 5). To specify a partial conformation, for each state, the regions between which base pairing is derived in the molecule is specified. Figure 4 illustrates a partial conformation for a state where the presence of two effector molecules did not activate the catalytic function of the molecule.

The conserved bases (indicated by bold wavy lines) are specified to explicitly bind to the helix II region when the two effectors are present, in order to inhibit the catalytic activity of the molecule. Aside from the fixed base pairs (E1 and E2 with their receptor sites, and the conserved CUGAUGAG-region with helix II), the remaining positions are not restricted, and can either form complementary base pairs with the remaining bases (i.e., part of helix) or completely unpaired in that particular state. Note that the randomisations of complementary positions are permitted.

Fixing the partial conformation beforehand seems to resemble the protocol suggested by [15]. However, since only partial regions in the conformation are fixed, in actual fact, the P-ERM protocol still maintains the degree of freedom in generating various structural configurations—rather than stereotyping the molecular structure into a predefined homogeneous conformation.

## Results and discussion

For the experiment, we created 10 different sets of partial conformation for each type of binary logic operator (T1 to T10). The difference between each set is the length of each element, which was randomly selected within the constraints detailed in Table 6. For each set, the partial conformations corresponding to the binary logic operations are explicitly defined. For instance, the partial conformation for any molecular gate where in one state, the presence of both effectors yields no activation of the ribozyme core is equivalent to the partial conformation presented for the XOR logic gate depicted in Figure 4. Compared to the random base pairing assignment suggested in P-ER2 protocol, the partial conformation contains mandatory base paired regions that must be preserved by *multiSrch*. In order to promote structural variability, the selection of the complementary pairings is randomised within the specified range (i.e., either a region in the hairpin loop region of helix II, or the helix II region itself).

**Table 6:** Design space for computational nucleic acids

**Type**	**Maximum no.**	**Length range**
Helix	-	4–15
Hairpin Loop	0–3	4–15
Internal Loop	0–3	2–8
Bulge	0–1	1–8
Junction	0–3	4–8
OBS	2	15–22
Linker	2	0–5

Design space for computational nucleic acids derived from potential candidates reviewed in [30]. As reported in our previous work [30], we have compiled a database of natural small catalytic nucleic acids and synthetically developed functional nucleic acids. Using this database, we have extracted the structural features that are common across these molecules. The column “Maximum no.” refers to the number of occurrences for each secondary structure element and the corresponding column “Length Range” represents the length for each element.

The parameter settings for *multiSrch* are shown in Table 7. The settings are largely based on the findings of the evaluation study to generate candidates for DNA and RNA gates described in [30]. The parameter settings are kept constant for each set of structural configurations. Because *multiSrch* is a deterministic algorithm, therefore only one run was required.

**Table 7:** **Default parameter settings for *multiSrch *[**[38]**]**

**Parameter**	**Value**
$D(\Psi^{*})$ Order	Descending
*Ξ*(*x*)	Eq. 5.7 with *E*_*gap*_=−6.0
*Λ*	300
KEEP	*Ξ*(*x*)−*min*(*Ξ*(*x*))=±5.0

Default parameter setting for *multiSrch*[38].

To allow for direct comparison with the previous results generated by the P-ER2 protocol, we divided our results into two different categories (LG-T and LG-B). These categories are differentiated by the initial state of its binary logic operations, similar to the initialisation strategy undertaken in the previous section. Table 8 shows the result for the LG-B logic operators (where without the presence of both effector molecules, the operators are inactive), while Table 9 shows the result for LG-T operators (where the operator would remain inactive, in the presence of both effector molecules). For each logic gate, 300 candidate sequences were generated based on the sets of partial conformations T1 to T10. These 300 candidate sequences were then filtered using the filter cascade shown in Table 4. In the inactive state, the conserved region must form base pairs with any regions from the helical arms II (helix II) until the hairpin loops next to the helix (includes both effector binding sites), and in the active state, this conserved region must be unpaired and the overall structure must have three helices [39,40] that resembles the conformation of an active hammerhead ribozyme.

**Table 8:** **Distribution of filtered candidate sequences generated by the P-ERM (cf. Table **5**) protocol for LG-B binary logical operators**

	**T1**	**T2**	**T3**	**T4**	**T5**	**T6**	**T7**	**T8**	**T9**	**T10**	**Rate**
A ∘_9_B	210	205	269	300	300	300	209	300	278	200	85.7%
A ∘_10_B	281	300	297	245	298	300	300	285	275	300	96.0%
A ∘_11_B	200	201	188	181	206	204	200	192	201	214	66.2%
A ∘_12_B	264	276	259	231	253	250	239	293	262	273	86.7%
A ∘_13_B	300	300	221	269	277	209	203	269	212	277	84.6%
A ∘_14_B	278	300	251	245	251	300	300	300	265	300	93.0%
A ∘_15_B	242	208	203	208	214	200	204	193	184	201	68.6%

Distribution of filtered candidate sequences generated by the P-ERM (cf. Table 5) protocol with *multiSrch* multi-stable states designer, classified according to the type of binary logic operator (LG-B) shown in the bottom half of Table 2. Depicted in the table is the number of candidates that passed the seven filter cascades (cf. Table 4) for each run. Before the filtering protocol, 300 candidates were generated for each type of logic operation in each run. The mean percentage of success rate is given in the “Rate” column.

**Table 9:** **Distribution of filtered candidate sequences generated by the P-ERM (cf. Table **5**) protocol for LG-T binary logical operators**

	**T1**	**T2**	**T3**	**T4**	**T5**	**T6**	**T7**	**T8**	**T9**	**T10**	**Rate**
A ∘_2_B	298	300	292	291	296	300	300	275	275	300	97.6%
A ∘_3_B	201	187	200	231	196	198	200	192	191	200	66.5%
A ∘_4_B	278	300	297	245	298	300	300	285	275	259	94.6%
A ∘_5_B	300	199	246	200	300	259	210	212	278	198	80.1%
A ∘_6_B	278	300	297	245	213	300	300	285	275	300	93.0%
A ∘_7_B	209	200	209	194	251	200	183	192	200	192	67.6%
A ∘_8_B	275	300	251	245	300	300	300	300	265	300	94.5%

Distribution of filtered candidate sequence generated by the P-ERM protocol with *multiSrch* multi-stable designer, classified according to the type of binary logic operator (LG-T) shown in the top half of Table 2. The columns (T1 to T10) represent the number of candidate sequences that passed the seven filter cascades in Table 4. For each logic operator, 300 candidate sequences were generated. The mean percentage of success rate is given in the “Rate” column.

**Figure 5:**
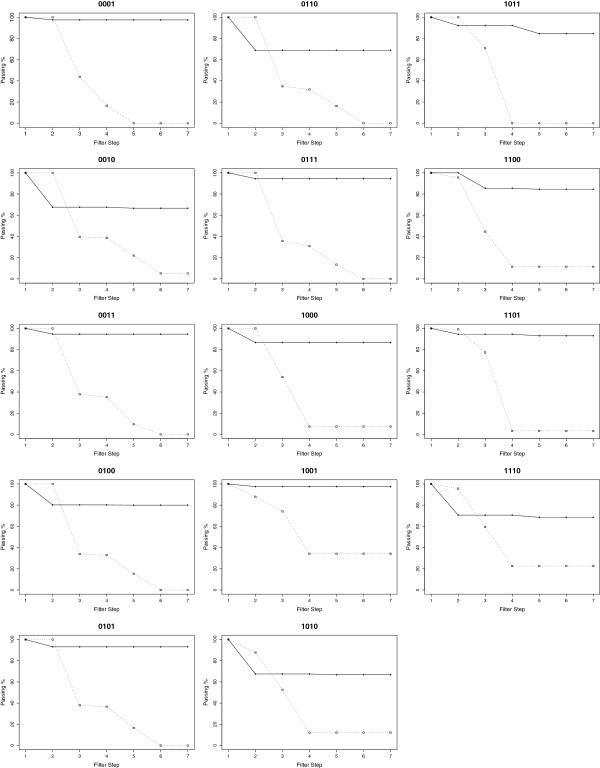
**Quality of the design of nucleic acid logic gates using the single-state and multi-state sequence designers.** Quality of the design of nucleic acid logic gates using the single-state and multi-state sequence designers. The graphs show the percentage of candidates that passed the filter for every stage in the filter described in Table 4. A Solid line represents candidates that were generated by multi-state designer, and a dashed line represents candidates from the single-state designer. The title of each graph represents from left to right, the output bits of the following input patterns (11, 10, 01, 00), with the inputs in the order of [input-B input-A], see Table 2. For instance, an OR gate is denoted as 1110 in the figure. Refer Tables 8 and 9 for the actual number of candidates.

The overall performance of the revised computational protocol with the addition of *multiSrch* is shown in Figure 5. In terms of the number of candidates that passed the filter cascade, the P-ERM protocol with *multiSrch* performed significantly better when compared to results from P-ER2 protocol. Compared to the low passing rates we observed for LG-B and LG-T candidate sequences that pass filter step 4 in P-ER2, majority of the candidate sequences generated by *multiSrch* in the P-ERM protocol (for both LG-B and LG-T) pass filter step 4. However, we observed that for A ∘_11_B (1010), OR (1110), A ∘_3_B (0010), and the XOR (0110) logic operators (cf. Figure 5), there is a slight dip of the candidate sequences in filter step 2. This indicates that the candidate sequences for the four logic operators mentioned, the active hammerhead ribozyme conformation was not obtained. The consistency of the results (for T1 to T10) is also maintained as shown in Tables 8 and 9.

For LG-B logic operators, (cf. Table 8), one can observe a significant improvement in the number of candidate sequences that pass the filtering procedure (indicated by percentage of passing in column Rate in Table 8) when compared to the P-ER2 protocol. Note the number of candidate sequences that pass the filter cascade. The worst passing rate across all types (T1 to T10) was recorded at ≈60%, which is still significantly better than the ≈25% observed in the previous section.

Analysis of the filtering process showed that the reduction of the candidate sequences occurred during filter step 2, where the conformation resembling an active hammerhead ribozyme was not obtained from the four meta-stable conformations. For instance, for A ∘_11_B gate with the output state of 1010, we found that the binding of a single effector molecule did not trigger any conformational shift that resembles an active ribozyme conformation.

Note that the filtering procedure conducted here is a direct implementation of the filtering model suggested by [15]. In order to test the presence of an active hammerhead ribozyme conformation, Penchovsky and Breaker in [15] suggested replacing the bases for the effector binding sites with “X”s due to the lack of multi-folding prediction programs. When one folds the sequence using *RNAfold*, this “X”s-region would represent a mandatory unpaired region thus simulating the conformation of a molecule with an effector molecule externally bound to it. Although we adopted the same filter technique, we do not think that this filter accurately predicts the inter-molecular binding between these interacting molecules. The amount of energy released during the formation of external binding is strong enough to break or shift existing internal hybridisation bonds and thus can lead to a change in conformation of the molecule. If we consider the intermolecular binding efficiency between the effector molecule and receptor site instead of the refolding of sequences with “X”s-region, then more than 90% of the generated sequences from *multiSrch* for LG-B have perfect binding between effector and receptor site as simulated by *RNAup*[41].

The reduction of candidate sequences for A ∘_11_B (1010) and OR (1110) logic gates in filter step 2, can also be contributed by the low free energy generated from these candidate sequences. For a low free energy structure, the base pair composition is highly dominated by either C‐G or G‐C pairing. Despite the effort to enforce the identical base pairing rules (cf. filter step 1 in Table 4), there is still an abundance of C‐G and G‐C pairings occurring (i.e., non-consecutive, but distributed in the group of three or four). By specifying the *ΔG*_*opt*_ target value in *multiSrch*, the algorithm is then able to generate sequences with higher free energy values which are better suited to the meta-stable molecules to be implemented [38]. For this purpose, *ΔG*_*opt*_ can be based on nucleic acids logic gates that have already been engineered in the laboratory.

Table 9 shows the overall result of generating candidate sequences for LG-T logic operators. The results are similar to the LG-B logic operators in Table 8, where there are two types of logic operators (A ∘_3_B and XOR) where the success rates are below ≈66%. During the filtering process, the reduction in candidate sequences occurs during filter step 2. Upon closer inspection, we found that the filtering procedure for step 2 in Table 4 that is implemented based on the model of [15] might be flawed because the base pairing formation between the effector molecules and its corresponding binding site are present when the generated sequences are simulated using *RNAup*[41]. The inability of the molecules to shift conformation (with the substitutions of “X”s bases) might be due to the low free energy of these sequences. The possibility to specify a desired MFE (*ΔG*_*opt*_) for the *multiSrch* algorithm was motivated by this issue.

From the results in Tables 8 and 9, the type gates with consistently poor numbers of filtered candidates across all ten structural designs (T1 to T10) were selected. Four gates were identified, the A ∘_11_B (1010), OR (1110), A ∘_3_B(0010), and the XOR (0110) gates where the success rate is less than ≈69%. The partial conformations for each of these gates can be classified as unfavourable. For these molecular gates, the presence of any one of the effectors can activate the ribozyme. However, the partial conformation that is arbitrarily selected in this comparison study is inadequate for dealing with this condition, because in the inactive state, some regions belonging to the receptor site always bind together. The self-assembly between the effector and receptor site for either one of the input (A or B), might not be sufficient in triggering a conformational change to activate the ribozyme. The design of A ∘_11_B logic gate would be more favourable if the effector representing input B is shorter compared to the effector for input A. The binding of effector B is estimated not to change the conformation as much as the binding of effector A to its receptor site. A favourable partial conformation design for each of these four gates would improve the passing rate of the candidate sequences especially for filter step two. The comparison study in [38] showed that the default setting of the *multiSrch* algorithm usually arrives at sequences with low MFE. These sequences are therefore quite stable and would require overcoming a high energy barrier to disassociate some of the existing base pairs.

A test to generate sequences for the four gates which were difficult to design using the same initial structures were conducted with a target MFE of *ΔG*_*opt*_= -40.00 kcal/mol. There is an increase in the percentage of success rate of all four logic gates, from ≈66% to ≈81% as indicated in Table 10. For the two logic gates of A ∘_11_B (1010), and OR (1110), the success rate increased up to ≈92%, approximately 10% better than the other two logic gates of A ∘_5_B (0100), and XOR (0110). The likely cause of this issue is the design of the partial conformation itself. For instance, in the design of XOR gate, the design of external binding for both effector molecules must be strong enough to completely disrupt the formation of an active conformation that needs to be present with a single effector molecule.

**Table 10:** Design of logic gates using a desired MFE

	**T1**	**T2**	**T3**	**T4**	**T5**	**T6**	**T7**	**T8**	**T9**	**T10**	**Rate**
A ∘_11_B	281	281	278	273	286	274	266	272	271	284	92.0%
A ∘_15_B	262	278	284	288	272	285	276	283	264	291	92.8%
A ∘_3_B	251	254	240	272	255	243	249	258	255	261	84.6%
A ∘_8_B	221	243	233	245	251	240	232	227	244	253	81.6%

Design of logic gates using a desired MFE. The number of candidates that pass the filter cascade generated for the four selected binary logic operators using *ΔG*_*opt*_ = -40.00 kcal/mol.

It is best, if we can supply the multi-stable states design algorithm (*multiSrch*) with complete structural details of the molecule, including changes in its meta-stable state. Regardless of the level of details available in the partial conformations, the computational protocol (P-ERM) should still be able to produce a good number of candidate sequences, as shown in this evaluation (Table 10). The success rate of the “difficult” structures illustrated by the XOR and A ∘_3_B are still above 80%, which is very encouraging because only partial conformations are supplied in the protocol. From the pool of successful candidate sequences, one can simply select any of the workable conformations as inputs for *multiSrch*, and accordingly with the estimation of MFE and energy gap, one would likely be able to produce a good number of candidate sequences.

Evidently, the task of designing the partial conformation and the parameter tuning would fall under the direction of the user. Using the knowledge of the structural conformation and the aid of the protocol, the user is able to design a better architecture of the nucleic acid unit and a better generation of candidate sequences to suit this architecture. In order to construct a fixed structural configuration, one is required to fix the mandatory base pairing and unpaired region of the molecules, thus creating a partial conformation (Figure 4) of the molecules for each meta-stable state. The undefined regions (i.e., non-mandatory base positions) are allowed to form base pair or remain unpaired as long as the mandatory base pairings and unpaired regions are preserved. Only the conserved bases are specified as constraint, allowing another level of diversity for the sequences. This approach is suitable if the target conformation is only partially known beforehand.

**Figure 6:**
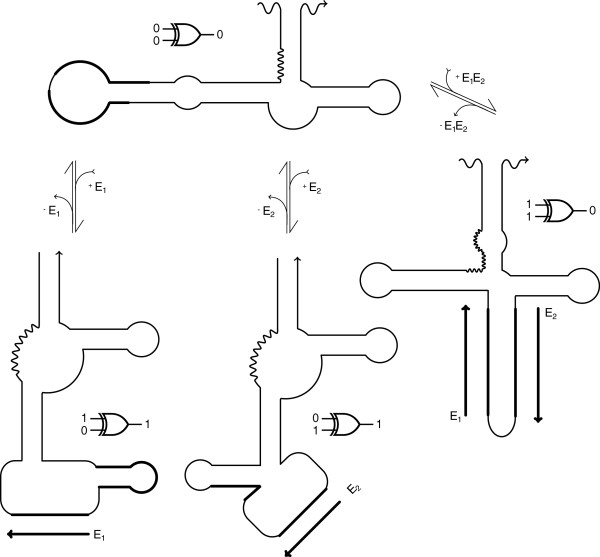
**Molecular XOR gate generated using P-ERM computational protocol.** Molecular XOR gate generated using P-ERM computational protocol (selected from successful candidates in T1). The inactive conformation of the molecule (top left) changes in the presence of effector molecule E_1_, to the active ribozyme conformation (bottom left). The presence of effector molecule E_2_ triggers a different conformation shift, that also activate the ribozymes (middle bottom). The presence of both effectors stretches out both binding regions and disrupts the formation of an active hammerhead motif (right).

A design for a molecular XOR gate was selected to illustrate its operation from the pool of candidate sequences that passed all the filter stages in Table 4. The four meta-stable conformations of this XOR design are shown in Figure 6. For the logic operation of the molecular XOR gate the presence of a single effector molecule would trigger conformational changes that form the active conformation of the ribozyme. The presence of both effector molecules in this case would stretch the binding site region, disrupting the formation of the hammerhead motif and thus deactivating the ribozyme.

## Conclusions

Penchovsky and Breaker [15] suggested a computational protocol to assist in the construction of ribonucleic acid logic gates. The protocol however, is restricted and specifically tuned only for generating sequences for allosterically controlled ribozymes imitating conventional logic gates. This limits the degree of freedom in generating various structural configurations. For constructing computational nucleic acids, structural variability is important because in the laboratory, depending on the physico-chemical environment, only some designs are practicable. By having a set of structural configurations, it is then possible to not only use the configuration that is workable under the given conditions, but at the same time, allows for the best design to be applied. This is also important because it allows one to investigate the type of structural complexity that might be required to solve certain information processing tasks. For instance, the design of a cascade of computing units (to relay or transform a signal) might require a large number of different structural configurations. The protocol of [15] becomes insufficient if one desires to construct a number of computational units.

The development of a computational protocol, which enables the construction of functional nucleic acids that can act as a substrate for information processing is the focus in this study. A new protocol called P-ERM was developed. In general, the P-ERM protocol comprises of two phases (Table 5), the construction of the partial conformation (cf. Figure 4) and the generation of the candidate sequences (using *multiSrch*) that conform to the partial conformation. The partial conformation allows users to specify both the structural and sequence constraints for each state. Using the binary logic operators as a test case, the P-ERM protocol generated a set of structural configurations with candidate sequences that have a high success rate during the filtering procedure. The feasibility of generating the complete set of binary logic operators in this study indicates both the effectiveness (in term of generating sequences with high success rate) and the efficiency (using only one run of *multiSrch*) of the protocol. To accomplish the task of constructing these nucleic acid computers, the protocol managed to produce a diverse set of structures and sequences based on the design constraints. Although, the *in-silico* construction of computational nucleic acids using the protocol does not guarantee their success in the laboratory, the protocol contributes in identifying possible candidate solutions for the actual implementation.

The application of nucleic acids in bioimmersive computation has the potential to open up interesting possibilities. For instance, using the strands of noncoding RNAs (ncRNA), one could try to develop regulatory units that harness their conformational switching (triggered after the introduction of an effector molecule, i.e., in this case, a short ncRNA) to create sticky ends that bind to a particular codon on mRNAs. The development of regulatory control points, such as a set of riboswitches or allosterically controlled nucleic acids is also possible. For instance, using short ncRNAs as input, the set of allosterically controlled nucleic acids can be activated when specific effector molecules are present and release short RNA strands that bind to a specific codon in mRNA thus blocking protein synthesis. Our computational protocol can support the design of nucleic acid computers that function as detection units, or the design of a network of regulatory control points. Smart drugs that can sense the internal state of cell and intervene in the intracellular regulatory mechanisms may come within reach [42] and engineered molecular control mechanisms that can be integrated into cells would be a powerful tool for life-science research [43].

Before the potential of these long-term aims can be realised many obstacles in the laboratory need to be tackled and much better computational design procedures are required. A crucial issue will be the prediction of the interactions within complex mixtures of molecules. At present folding simulators for multiple interacting RNA strands are at their infancy and simulation tools capable of predicting DNA-RNA interactions do not exist. There is a need for a general methodology and supporting computational tools to create purpose-designed sets of interacting computational nucleic acids. This *in-silico*-first approach will enable designers to specify the physiological conditions plus additional constraints that should aid in the construction of well-defined computational units, and reduce the cost and time required in the laboratory.

## Competing interests

The authors declare that they have no competing interests.

## Authors’ contributions

EIR designed and implemented the computational protocol. KPZ provided the direction and guidance to the project and all authors contributed to, and have read and approved the final manuscript.
